# Endoscopic resection in treatment of intramural esophageal cysts: Retrospective analysis of 67 cases

**DOI:** 10.1055/a-2781-5586

**Published:** 2026-02-12

**Authors:** Shaobin Luo, Zu-Qiang Liu, Li Wang, Yi-Qun Zhang, Wei-Feng Chen, Lili Ma, Jian-Wei Hu, Ming-Yan Cai, Quan-Lin Li, Ping-Hong Zhou

**Affiliations:** 1 92323 Zhongshan Hospital Fudan University, Shanghai, China

**Keywords:** Endoscopy Upper GI Tract, Endoscopic resection (ESD, EMRc, ...), POEM, Barrett's and adenocarcinoma

## Abstract

**Background and study aims:**

Intramural esophageal cysts (ECs) are rare congenital malformations. This study aimed to investigate clinical characteristics of intramural ECs and evaluated safety and efficacy of endoscopic resection.

**Patients and methods:**

From August 2012 to June 2024, 67 patients with intramural ECs treated at the Endoscopy Center of Zhongshan Hospital were retrospectively analyzed. Data on baseline characteristics, clinical outcomes, and follow-up were collected.

**Results:**

Twenty-nine patients (17 males, mean age 49.8 years) underwent submucosal tunneling endoscopic resection (STER) and 38 patients (26 males, mean age 53.0 years) underwent endoscopic submucosal dissection (ESD) for intramural ECs. Mean specimen sizes were 2.8 ± 0.9 cm and 1.1 ± 0.6 cm, respectively ( *P* < 0.001). The STER group showed longer operative times (51.2 ± 20.6 vs. 32.6 ± 17.5 minutes, *P* < 0.001) and slower operation speed (0.13 ± 0.09 cm ^2^ /min vs. 0.21 ± 0.14 cm ^2^ /min, *P* = 0.032) compared with the ESD group. Complete resection rates for the STER and ESD groups were 82.8% and 94.7%, respectively ( *P* = 0.127). No major adverse events occurred in the groups. Complete resection was achieved in seven cases with extraluminal growth in the STER group without serious complications. During follow-up (median 37 months and 46 months, respectively), no local recurrence or strictures were observed in either group.

**Conclusions:**

Endoscopic resection of intramural ECs is safe and effective with fairly good long-term follow-up outcomes. The STER technique has advantages of completely resecting intramural esophageal cysts originating from the deep muscularis propria layer, particularly lesions with extraluminal growth.

## Introduction

Esophageal cysts (ECs), although rare with an estimated incidence of 1:50,000, represent clinically significant due to their potential for symptom escalation and anatomic complications 1 . According to their anatomical location, esophageal cysts are divided into intramural cysts (located in the wall of the esophagus, with connection to the submucosa or muscularis) and mediastinal cysts (adjacent to the esophagus but growing independently) 2 . These congenital or acquired lesions, classified as duplication cysts, bronchogenic cysts, or inclusion cysts, predominantly present in the middle and lower esophagus 3 . Although small cysts (< 3 cm) often remain asymptomatic, larger lesions cause dysphagia, chest pain or compression of the respiratory tract, and even complications including infection, bleeding, or rupture 4 . Bronchogenic cysts are congenital malformations derived from the primitive foregut with abnormal budding 5 . They may occur in the mediastinum and pericardium, but are rarely reported in the esophagus 5 .

Asymptomatic small cysts can be followed up and surgical resection is indicated for patients with symptoms, large volume, or uncertain diagnosis. Traditional surgery strategies achieve complete resection rates of 85% to 90% but carry substantial morbidity including pneumonia, anastomotic leaks, and prolonged hospitalization 6 . With significant advances in endoscopic technologies, therapeutic endoscopic techniques have become potential alternatives for removing intramural esophageal cysts. Minimally invasive endoscopic approaches, such as cyst fenestration or needle aspiration, reduce procedure trauma but suffer from high recurrence rates due to incomplete cyst wall removal 7 . Endoscopic submucosal dissection (ESD) is an effective strategy for tumors originating from the muscularis propria and submucosa. Nevertheless, it is risky and challenging to use ESD to completely resect tumors originating from the deep muscularis propria (MP) layer, particularly those growing outside the esophageal wall 8 . Submucosal tunneling endoscopic resection (STER) is a combination of ESD with peroral endoscopic myotomy techniques 8 . Compared with conventional ESD, STER can resect lesions originating from the muscularis propria, while maintaining superficial mucosa integrity and reducing risk of postoperative esophageal leakage and secondary infection 8 . This therapeutic dilemma is exacerbated in high-risk populations of elderly patients.

Currently, most of these lesions have been reported as individual cases 9 10 . Differences in reports on the clinical features of ECs and some controversies about the treatment strategy for ECs exist. Notably, ECs with an extraluminal growth pattern pose unique diagnostic and therapeutic challenges due to their obscured endoscopic visualization. In this study, we analyzed characteristics of intramural ECs and evaluated efficacy and safety of endoscopic resection for intramural ECs.

## Patients and methods

### Patients

From August 2012 to June 2024, 67 patients with intramural ECs underwent ER at the Endoscopy Center and Endoscopy Research Institute, Zhongshan Hospital (Supplementary Figure 1). All patients underwent endoscopic resection by experienced endoscopists (> 500 ESD/STER cases). The study protocol was approved by the Ethics Committee of Zhongshan Hospital and was in accordance with the Declaration of Helsinki. Consent forms were secured from each participant prior to their involvement in the study.

### Endoscopic resection procedures

The procedure of STER for esophageal cyst with extraluminal growth pattern.Video 1

**Fig. 1:**
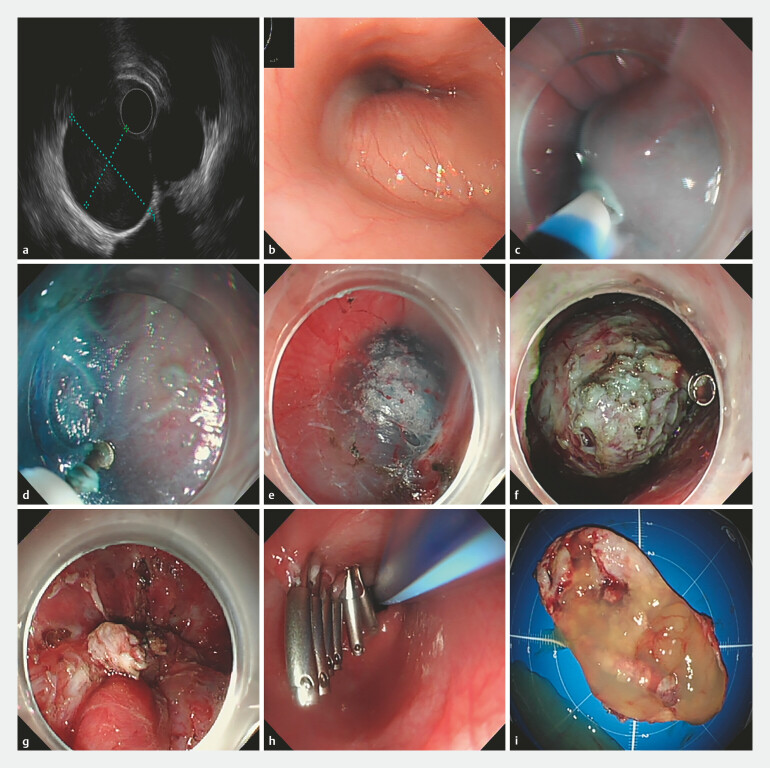
The procedure of STER for a cardial cyst with extraluminal growth pattern. **a** Endoscopic ultrasound confirmed that the cysts in the cardia originated from the muscularis propria layer with extraluminal growth pattern. **b** A smooth hemispherical submucosal lesion in the cardia. **c** The submucosal injection. **d,e** Submucosal dissection exposed a soft cyst. **f** The cyst wall was completely dissected. **g** Hemostasis was ensured with thermal coagulation showed no active bleeding. **h** The tunnel entrance was closed by metal clips and a gastric tube was placed. **i** The resected specimen (3.5 cm×3.5 cm).

**Fig. 2:**
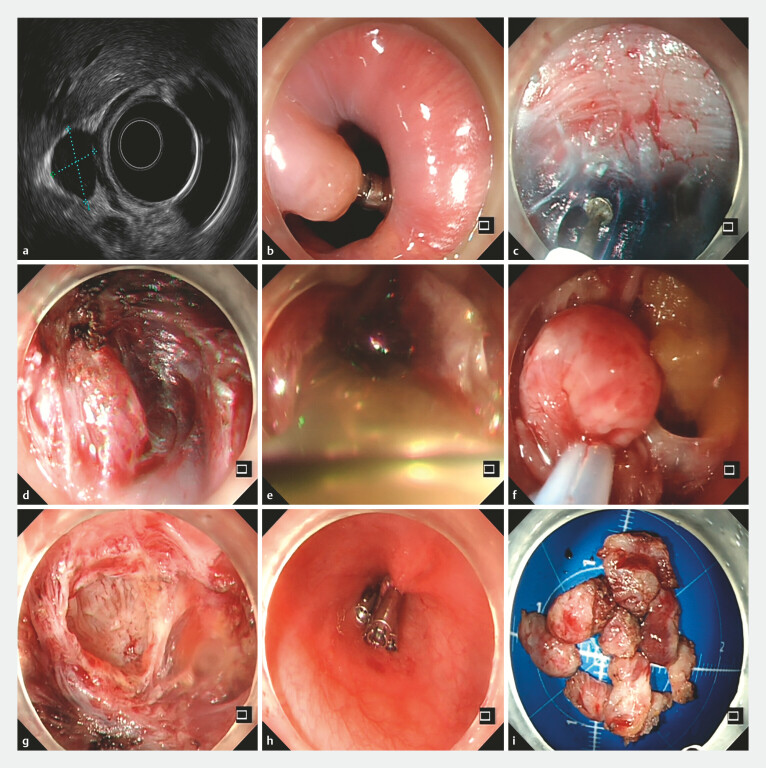
The procedure of STER for a lower esophageal cyst with extraluminal growth pattern. **a** Endoscopic ultrasound confirmed that the cysts in the lower esophagus originated from the muscularis propria layer with extraluminal growth pattern. **b** A smooth hemispherical submucosal lesion in the lower esophagus. **c** The submucosal injection. **d** Submucosal dissection exposed a soft extra-luminal cyst. **e** The cyst wall was incised and release cloudy milky fluid. **f** The cyst wall was completely dissected. **g** Hemostasis was ensured with thermal coagulation showed no active bleeding. **h** The tunnel entrance was closed by metal clips. **i** The resected specimen (2.0 cm×1.8 cm).

The procedure was performed under general anesthesia with tracheal intubation by using a gastroscope (GIF-Q290J, Olympus, Tokyo, Japan). Preoperative computed tomography (CT) and endoscopic ultrasound (EUS) were routinely performed to assess cyst location, layer of origin, and relationship with surrounding tissues. The STER procedure was performed as follows ( Video 1 ). Submucosal injection was performed. A 2-cm longitudinal mucosal incision was made 5 cm above the lesion site with an IT knife (Olympus, Tokyo, Japan). A tunnel was established along the submucosa until the lesion was exposed. When the cyst was exfoliated, the surrounding connective tissue was carefully excised to ensure that the cystic wall was as intact as possible. If the intraoperative resection was not completely performed, the cystic wall was incised, fluid in the cyst was sufficiently suctioned, and remaining lesions were ablated using argon plasma coagulation (APC). The tunnel opening and mucosal damage were closed with titanium clips ( Fig. 1 and Fig. 2 ).

**Fig. 3:**
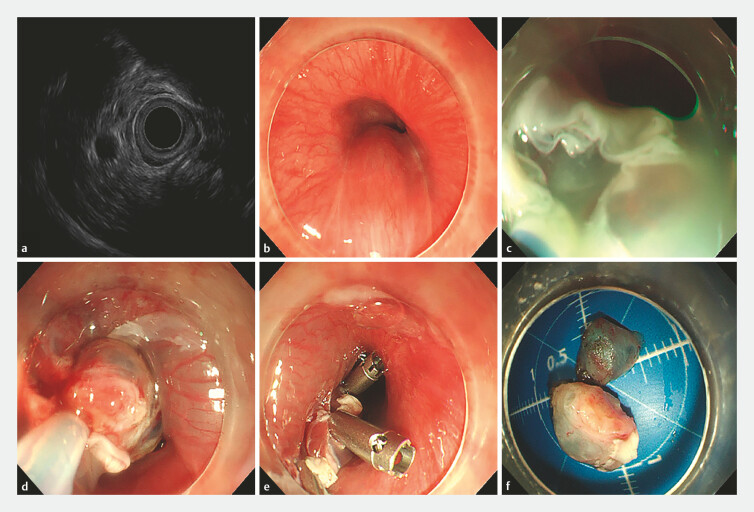
The procedure of ESD for an esophageal cyst. **a** Endoscopic ultrasound confirmed that the cysts in the esophagus originated from the submucosal layer. **b** A smooth hemispherical submucosal lesion in the upper esophagus. **c** Submucosal injection. **d** After incision of the muscular layer, the cyst wall was completely dissected. **e** The tunnel entrance was closed by metal clips. **f** The resected specimen (1.8 cm×1.6 cm).

The ESD procedure was done as follows. Local submucosal injection showed positive mucosal lifting sign on the lesion surface. The IT knife was used to cut the mucosal layer at the edge of the cystic wall along the lateral side of the labeled point layer by layer along with the submucosal layer, then the cyst was completely stripped. During the operation, hemostatic forceps were used on suspected bleeding and vascular exposure, and the wound was treated. Titanium clips were used to close the wound at the deeper part of it ( Fig. 3 ).

### Postoperative management and Follow-up

Patients were fasted for 48 hours after the operation, intravenous antibiotics and parenteral nutrition were administered, and basic vital signs were monitored. Postoperative complications, such as elevated body temperature, hemoptysis, chest pain, dyspnea, and abdominal pain, were monitored. Endoscopic follow-up was recommended for patients at 3, 6, and 12 months after surgery and once a year after surgery through gastroscopy to check wound healing and identify presence of residual or recurrent lesions. Chest CT was recommended once a year. If CT abnormalities were found during follow-up, EUS examination was performed to determine lesion characteristics and origins.

### Statistical analysis

Data were analyzed with SPSS software (Version 22.0, IBM SPSS). Continuous variables are described as means ± standard deviations, whereas categorical variables are described as frequencies and percentages. The Student’s t-test or Mann-Whitney U test was used to compare continuous variables and the chi-square test or Fisher’s exact test was used to compare categorical variables. Statistical significance was defined as p-values < 0.05.

## Results

### Baseline characteristics and EUS findings

Table 1 shows significant differences in baseline characteristics between the STER and ESD groups. Twenty-nine patients (17 males, mean age 49.8 years) underwent STER and 38 patients (26 males, mean age 53.0 years) underwent ESD for intramural ECs. larger tumors size (2.8 ± 0.9 cm vs. 1.1 ± 0.6 cm, *P* < 0.001). Symptomatic presentation differed substantially, with dysphagia (37.9% vs. 2.6%, *P* < 0.001) and regurgitation (27.6% vs. 5.3%, *P* = 0.012) being more common in the STER group, whereas asymptomatic cases were prevalent in the ESD cohort (73.7% vs. 13.8%, *P* < 0.001). EUS findings further distinguished the groups. STER lesions predominantly invaded the muscularis propria (96.6% vs. 5.3%, *P* < 0.001) and exhibited extraluminal growth (24.1% vs. 0.0%, *P* = 0.002), whereas ESD lesions were confined to the submucosa (94.7% vs. 3.4%, *P* < 0.001). No significant differences were observed in gender, cyst types, echostructure, or homogeneity ( *P* > 0.05).

**Table:** **Table 1** Baseline characteristics and EUS findings.

Baseline characteristics	STER (n = 29)	ESD (n = 38)	*P* value
Age, year	49.8 ± 15.8	53.0 ± 13.2	0.368
Male, n (%)	17 (58.6%)	26 (68.4%)	0.408
Tumor size (cm)	2.8 ± 0.9	1.1 ± 0.6	< 0.001
Cyst types, n (%)			0.712
Duplication cysts	22 (75.9%)	30 (78.9%)	-
Bronchogenic cysts	3 (10.3%)	5 (13.2%)	-
Inclusion cyst	4 (13.8%)	3 (7.9%)	-
Lesion location, n (%)			0.664
Upper esophagus	4 (13.8%)	9 (23.7%)	-
Middle esophagus	7 (24.1%)	5 (13.2%)	-
Lower esophagus	17 (58.6%)	21 (55.3%)	-
Cardia	1 (3.4%)	3 (7.9%)	-
Symptoms, n (%)			
Asymptomatic	4 (13.8%)	28 (73.7%)	<0.001
Dysphagia	11 (37.9%)	1 (2.6%)	<0.001
Regurgitation	8 (27.6%)	2 (5.3%)	0.012
Epigastric discomfort	6 (20.7%)	7 (18.4%)	0.817
Duration of symptom, years	1.6±1.9	2.3±1.7	0.102
Surface, n (%)			
Smooth lucency	26 (89.7%)	36 (94.7%)	0.657
Slight concavity	3 (10.3%)	2 (5.3%)	0.642
**EUS findings**			
Depth of invasion, n (%)			
Muscularis propria	28 (96.6%)	2 (5.3%)	< 0.001
Submucosa	1 (3.4%)	36 (94.7%)	<0.001
Echostructure, n (%)			
Anecho	3 (10.3%)	4 (10.5%)	1.000
Hypoechoic	26 (89.7%)	34 (89.5%)	1.000
Extraluminal growth, n (%)	7 (24.1%)	0 (0%)	-
Intracystic septa, n (%)	2 (6.9%)	3 (7.9%)	1.000
ESD, endoscopic submucosal dissection; EUS, endoscopic ultrasound; STER, submucosal tunnel endoscopic resection.			

### Procedure data and follow-up

Procedure data and follow-up outcomes are shown in Table 2 . The STER group had longer operative times (51.2 ± 20.6 vs. 32.6 ± 17.5 minutes, *P* < 0.001) and slower operation speed (0.13 ± 0.09 cm ^2^ /min vs. 0.21 ± 0.14cm ^2^ /min, *P* = 0.032) compared with the ESD group. Complete resection rates for cyst walls did not differ statistically (94.7% vs. 82.8%, *P* = 0.127). Cystic fluid characteristics were similar between groups, with muddy and sticky fluid being predominant (75.9% vs. 71.1%, *P* = 0.656). Adverse events (AEs) included subcutaneous emphysema (4 cases), delayed bleeding (1 case), febrile episode (1 case), pulmonary inflammation (1 case) in the STER group. During follow-up, no local recurrence or strictures were observed in either group.

**Table:** **Table 2** Procedure data and follow-up.

Procedure data	STER (n = 29)	ESD (n = 38)	*P* value
Procedure duration, min	51.2 ± 20.6	32.6 ± 17.5	< 0.001
Operation speed, cm ^2^ /min	0.13 ± 0.09	0.21 ± 0.14	0.032
Complete resection of cyst wall, n (%)	24 (82.8%)	36 (94.7%)	0.127
Cystic fluid, n (%)			
Clear and transparent	6 (20.7%)	11 (28.9%)	0.429
Muddy and sticky	22 (75.9%)	27 (71.1%)	0.656
Chocolate-like	1 (3.4%)	0 (0.0%)	-
Damage to the muscle layer, n (%)	2 (6.9%)	0 (0.0%)	-
Nasogastric tubes, n (%)	5 (17.2%)	3 (7.9%)	0.284
Adverse events, n (%)	7 (24.1%)	1 (2.6%)	0.017
Delayed bleeding	1 (3.4%)	0 (0.0%)	-
Febrile episode (> 38.5°C)	1 (3.4%)	1 (2.6%)	-
Subcutaneous emphysema	4 (13.8%)	0 (0.0%)	-
Pulmonary inflammation	1 (3.4%)	0 (0.0%)	-
Hospital stay, days	2.1 ± 0.8	2.0 ± 0.9	0.621
Follow-up, months	37 (6–93)	46 (19–158)	0.058
Local recurrence, n (%)	0 (0.0%)	0 (0.0%)	-
Stricture, n (%)	0 (0.0%)	0 (0.0%)	-
ESD, endoscopic submucosal dissection; STER, submucosal tunnel endoscopic resection.			

### Clinical features and outcomes of lesions with extraluminal growth in STER group

Analysis of seven cases with extraluminal growth in STER group is shown in Table 3 . Lesions were located in the upper esophagus (1 case), middle esophagus (3 cases), and lower esophagus (2 cases), with one case located in the cardia. Tumor sizes ranged from 2.3 × 1.0 cm to 5.0 × 2.9 cm, with duplication cysts being the most common type (5/7 cases), followed by one bronchogenic cyst and one inclusion cyst. Symptoms included dysphagia (3 cases), epigastric discomfort (2 cases), regurgitation (1 case), and one asymptomatic presentation. Morphologically, lesions exhibited nodular (3 cases), stripe-like (3 cases), or cylindrical (1 case) configurations. All tumors invaded the MP, and displayed clear margins.

**Table:** **Table 3** Clinical features and outcomes of cases with extraluminal growth in STER group.

Case	Age	Sex	Position	Tumor size (cm)	Cyst type	Symptom	Depth of invasion
1	40	F	Cardia	3.5×2.0	Duplication cysts	Dysphagia	MP
2	39	F	Lower	3.5×2.3	Duplication cysts	Dysphagia	MP
3	31	M	Lower	4.6×2.1	Duplication cysts	Regurgitation	MP
4	68	M	Middle	3.0×2.0	Duplication cysts	Epigastric discomfort	MP
5	25	F	Upper	2.3×1.0	Bronchogenic cysts	Epigastric discomfort	MP
6	47	M	Middle (Adjacent to aorta)	5.0×2.9	Inclusion cyst	Dysphagia	MP
7	58	F	Middle	2.0×1.2	Duplication cysts	Asymptomatic	MP
**Case**	**Procedure duration (min)**	**Complete resection**	**Mucosal injury**	**Muscle layer injury**	**Nasogastric tube**	**Adverse event**	**Hospital stay (days)**
1	60	Yes	No	No	Yes	Subcutaneous emphysema	3
2	70	Yes	No	No	Yes	No	2
3	62	Yes	No	No	No	No	2
4	50	Yes	No	No	No	Subcutaneous emphysema	3
5	54	Yes	No	No	Yes	No	2
6	85	Yes	No	No	Yes	No	3
7	55	Yes	No	No	No	No	2
F, female; M, male; MP, muscularis propria layer; STER, submucosal tunneling endoscopic resection.							

Procedurally, operative durations varied from 50 to 85 minutes (median: 60 minutes). Complete resection was achieved in all cases. No mucosal or muscle layer injury occurred in any of the cases. Nasogastric tubes were utilized in four patients (57.1%). AEs included subcutaneous emphysema in two cases (28.6%), with no other complications reported. Hospital stays were brief, lasting 2 to 3 days (median: 2 days).

## Discussion

Esophageal submucosal lesions, particularly large intramural cysts, are clinically challenging entities due to their insidious growth patterns and potential for severe complications 11 . These lesions typically manifest with nonspecific symptoms such as dysphagia, retrosternal pain, and gastroesophageal reflux disease (GERD), although asymptomatic presentations are not uncommon, delaying diagnosis until cyst enlargement compresses adjacent mediastinal structures 12 . Histopathologically, these cysts are characterized by a stratified squamous or columnar epithelial lining embedded within the MP, a feature that complicates conventional endoscopic resection due to the high risk of perforation and mediastinal contamination 12 .

Accurate diagnosis of esophageal cysts hinges on a combination of clinical suspicion, imaging modalities, and histopathologic correlation, with EUS emerging as the standard for preoperative assessment 13 . EUS provides high-resolution, layer-by-layer visualization of the esophageal wall, enabling precise differentiation between cystic and solid submucosal lesions 14 . This capability is critical, given that intramural esophageal lesions initially misclassified as solid tumors on CT are later confirmed as cysts via EUS, underscoring its diagnostic superiority. Characteristic EUS features of esophageal cysts include an anechoic or hypoechoic lumen with posterior acoustic enhancement, a well-defined smooth border, and origination from the muscularis propria (third layer) or submucosa (second layer) 15 . In addition, EUS-guided fine-needle aspiration allows safe sampling of cyst fluid for biochemical and cytological analysis, distinguishing true epithium-lined cysts from mimics such as abscesses or necrotic tumors 16 . The stratified imaging provided by EUS not only confirms cyst etiology but also informs therapeutic decision-making by mapping lesion depth and proximity to critical structures. For instance, cysts confined to the MP are prime candidates for STER, whereas those involving the adventitia or abutting major vessels may require multidisciplinary approaches 17 . Furthermore, EUS identifies high-risk features predictive of procedure complexity, including cyst wall calcifications, septations, and vascularity 13 . Importantly, EUS also rules out contraindications to submucosal tunneling, such as extensive submucosal fibrosis or overlapping malignancies 18 . Despite its advantages, EUS interpretation requires expertise to avoid pitfalls. For example, cysts with viscous or proteinaceous contents may display heterogeneous echogenicity, mimicking solid gastrointestinal stromal tumors (GISTs) 19 .

Although endoscopic resection has emerged as a minimally invasive alternative to surgery, its use remains limited for deep-seated lesions 20 . Endoscopic full-thickness resection (EFTR) necessitates intentional full-thickness resection, which inadvertently increases postoperative infection risks and mandates prolonged hospitalization for prophylactic antibiotic administration 21 . In this study, STER has revolutionized management of esophageal cysts by combining principles of natural orifice transluminal endoscopic surgery with precise submucosal dissection 22 . The cornerstone of STER lies in creation of a submucosal tunnel starting 3 to 5 cm proximal to the lesion, which allows for en bloc resection of the cyst wall while preserving the overlying mucosal layer 22 . This approach mitigates two critical limitations of traditional techniques. First, the intact mucosal barrier significantly reduces incidence of mediastinal infection by preventing direct exposure of the mediastinum to gastrointestinal flora 22 . Second, the submucosal tunnel facilitates meticulous dissection along the cyst pseudocapsule, minimizing residual lesion remnants—a common cause of recurrence in conventional endoscopic therapies 23 .

In the present study, complete resection rates for the cyst wall were 94.7% in the ESD group and 82.8% in the STER group. Results have shown no significant difference in complete resection rate between ESD and STER. However, when the endoscope enters submucosal tunnel, it has little effect on respiration during the procedure 24 . In addition, a good field of view during the procedure contributes to more accurate operation. Although no residual tumor or recurrence was noted during follow-up, the application of piecemeal resection for ECs should be carefully taken into consideration to ensure the best oncologic results. Anatomic preservation inherent to STER further mitigates recurrence risks by maintaining physiologic esophageal motility. Surgical cyst enucleation, although effective for giant cysts (> 10 cm), frequently necessitates myotomy or partial esophageal wall resection, creating fibrotic niches that predispose to pseudocyst formation 25 . The submucosal tunneling technique of STER circumvents these pitfalls by enabling cyst pseudocapsule-directed dissection 23 .

No massive bleeding, delayed perforation, or other severe complications occurred during or after the procedure. Delayed bleeding occurred in one patient in the STER group, which was treated with endoscopic irrigation to remove the clot, hot biopsy forceps, cauterization of the metal clamp, and gastric tube decompression. The STER group had larger tumors size and higher rates of MP invasion. Notably, the largest lesion (5.0 × 2.9 cm) was adjacent to the aorta but was resected without procedure complications. These outcomes underscore the feasibility of STER for extraluminal growths, despite technical challenges reflected in variable en bloc resection rates and occasional subcutaneous emphysema. A long surgical time was a risk factor for ER-related AEs, which is consistent with our results 26 . On the one hand, STER requires additional creation of a submucosal tunnel that provides space for endoscopic dissection, which is technically challenging. On the other hand, some studies suggested that the large tumors may be associated with endoscopic vision 27 . Crucially, the submucosal tunneling of STER acts as a biologic barrier, reducing bacterial translocation 28 . STER also outperforms ESD in preventing delayed perforation, attributable to stabilized dissection angles within the tunnel that minimize lateral thermal injury 25 . This preservation of mucosal integrity also mitigates GERD 29 .

Application of submucosal tunneling endoscopic resection (STER) for extraluminal lesions presents significant technical complexities. Extraluminal lesions lack intraluminal reference markers, necessitating precise preprocedure EUS mapping and real-time balloon-assisted localization (e.g., tracheal cannula balloon inflation) 30 . Although STER excels in resecting intramural cysts confined to the muscularis propria, its efficacy diminishes for lesions penetrating the adventitia or those with extraluminal extensions, whereas the ability of EFTR to achieve full-thickness excision becomes indispensable 30 . STER safeguards the mediastinum from contamination, whereas EFTR ensures complete en bloc removal of transmural or paraesophageal cysts. Anatomical precision is paramount in STER-EFTR, particularly for cysts abutting critical mediastinal structures. Preoperative planning with contrast-enhanced EUS and three-dimensional (3D) CT reconstruction enables precise mapping of cyst margins relative to the aorta, trachea, and pericardium 31 .

During dissection, the submucosal tunnel serves as a working corridor, allowing controlled EFTR under direct endoscopic visualization 32 . Notably, the combined technique reduces reliance on laparoscopic assistance, which is traditionally required for extraluminal cyst management. Proximity to the MP or mediastinal structures increases risk of unintentional deep muscle layer entry or transmural perforation during tunneling 33 . In our study, controlled submucosal injection created a “safety cushion,” whereas axial alignment adjustments maintained dissection within the submucosal plane, avoiding vascular and adjacent structures. Meticulous hook-knife incision, blunt dissection under negative-pressure suction, and piecemeal resection were employed. Limited extraluminal operating space and obscured visual fields required advanced 3D spatial awareness to prevent capsular rupture 34 . Extraluminal manipulation risks mediastinal emphysema or infection. Strict aseptic techniques, regulated CO _2_ insufflation pressure, and postoperative gastric tube decompression were critical. Early fasting and proton pump inhibitor therapy prevented esophageal leakage and reflux-related complications 35 .

There are several limitations to this study. First, as a single-center retrospective analysis, this study may have an element of selection bias. Second, the number of cases enrolled in our study was insufficient, and we were unable to confirm whether the symptoms were related to the intramural ECs themselves. Furthermore, management of infected esophageal cysts poses unique clinical challenges, such as whether STER can be applied to infectious esophageal cysts.

## Conclusions

In conclusion, our results indicate that endoscopic resection of intramural ECs is feasible, safe, and effective with fairly good long-term follow-up outcomes. Furthermore, appropriate preoperative diagnosis and careful selection of the treatment strategy based on lesion characteristics is critical for achieving optimal outcomes and minimizing complications. In future, prospective multicenter studies are needed to further evaluate the efficacy, safety, and long-term outcomes of endoscopic resection of intramural ECs.
